# Cerebral blood flow is associated with plasma and PET biomarkers of tau pathology in middle age

**DOI:** 10.1093/braincomms/fcaf249

**Published:** 2025-06-19

**Authors:** Alexander L Houck, Mohamad J Alshikho, Patrick J Lao, Jeffrey D Pyne, Indira C Turney, Jessica Mazen, Andrea Benavides, Christiane Hale, Mathieu Herman, Joncarlos Berroa, Natalie C Edwards, Jose Gutierrez, Sabrina Simoes, Jennifer J Manly, Adam M Brickman

**Affiliations:** Taub Institute for Research on Alzheimer’s Disease and the Aging Brain, Vagelos College of Physicians and Surgeons, Columbia University, New York, NY 10032, USA; Gertrude H. Sergievsky Center, Vagelos College of Physicians and Surgeons, Columbia University, New York, NY 10032, USA; Department of Neurology, Vagelos College of Physicians and Surgeons, Columbia University, New York, NY 10032, USA; Taub Institute for Research on Alzheimer’s Disease and the Aging Brain, Vagelos College of Physicians and Surgeons, Columbia University, New York, NY 10032, USA; Gertrude H. Sergievsky Center, Vagelos College of Physicians and Surgeons, Columbia University, New York, NY 10032, USA; Department of Neurology, Vagelos College of Physicians and Surgeons, Columbia University, New York, NY 10032, USA; Taub Institute for Research on Alzheimer’s Disease and the Aging Brain, Vagelos College of Physicians and Surgeons, Columbia University, New York, NY 10032, USA; Gertrude H. Sergievsky Center, Vagelos College of Physicians and Surgeons, Columbia University, New York, NY 10032, USA; Department of Neurology, Vagelos College of Physicians and Surgeons, Columbia University, New York, NY 10032, USA; Taub Institute for Research on Alzheimer’s Disease and the Aging Brain, Vagelos College of Physicians and Surgeons, Columbia University, New York, NY 10032, USA; Gertrude H. Sergievsky Center, Vagelos College of Physicians and Surgeons, Columbia University, New York, NY 10032, USA; Department of Neurology, Vagelos College of Physicians and Surgeons, Columbia University, New York, NY 10032, USA; Laboratory of Epidemiology & Population Sciences, National Institute on Aging, National Institutes of Health, Baltimore, MD 21224, USA; Taub Institute for Research on Alzheimer’s Disease and the Aging Brain, Vagelos College of Physicians and Surgeons, Columbia University, New York, NY 10032, USA; Gertrude H. Sergievsky Center, Vagelos College of Physicians and Surgeons, Columbia University, New York, NY 10032, USA; Department of Neurology, Vagelos College of Physicians and Surgeons, Columbia University, New York, NY 10032, USA; Taub Institute for Research on Alzheimer’s Disease and the Aging Brain, Vagelos College of Physicians and Surgeons, Columbia University, New York, NY 10032, USA; Gertrude H. Sergievsky Center, Vagelos College of Physicians and Surgeons, Columbia University, New York, NY 10032, USA; Department of Neurology, Vagelos College of Physicians and Surgeons, Columbia University, New York, NY 10032, USA; Taub Institute for Research on Alzheimer’s Disease and the Aging Brain, Vagelos College of Physicians and Surgeons, Columbia University, New York, NY 10032, USA; Gertrude H. Sergievsky Center, Vagelos College of Physicians and Surgeons, Columbia University, New York, NY 10032, USA; Department of Neurology, Vagelos College of Physicians and Surgeons, Columbia University, New York, NY 10032, USA; Taub Institute for Research on Alzheimer’s Disease and the Aging Brain, Vagelos College of Physicians and Surgeons, Columbia University, New York, NY 10032, USA; Department of Neurology, Vagelos College of Physicians and Surgeons, Columbia University, New York, NY 10032, USA; Taub Institute for Research on Alzheimer’s Disease and the Aging Brain, Vagelos College of Physicians and Surgeons, Columbia University, New York, NY 10032, USA; Gertrude H. Sergievsky Center, Vagelos College of Physicians and Surgeons, Columbia University, New York, NY 10032, USA; Department of Neurology, Vagelos College of Physicians and Surgeons, Columbia University, New York, NY 10032, USA; Taub Institute for Research on Alzheimer’s Disease and the Aging Brain, Vagelos College of Physicians and Surgeons, Columbia University, New York, NY 10032, USA; Gertrude H. Sergievsky Center, Vagelos College of Physicians and Surgeons, Columbia University, New York, NY 10032, USA; Department of Neurology, Vagelos College of Physicians and Surgeons, Columbia University, New York, NY 10032, USA; Department of Neurology, Vagelos College of Physicians and Surgeons, Columbia University, New York, NY 10032, USA; Taub Institute for Research on Alzheimer’s Disease and the Aging Brain, Vagelos College of Physicians and Surgeons, Columbia University, New York, NY 10032, USA; Department of Neurology, Vagelos College of Physicians and Surgeons, Columbia University, New York, NY 10032, USA; Taub Institute for Research on Alzheimer’s Disease and the Aging Brain, Vagelos College of Physicians and Surgeons, Columbia University, New York, NY 10032, USA; Gertrude H. Sergievsky Center, Vagelos College of Physicians and Surgeons, Columbia University, New York, NY 10032, USA; Department of Neurology, Vagelos College of Physicians and Surgeons, Columbia University, New York, NY 10032, USA; Taub Institute for Research on Alzheimer’s Disease and the Aging Brain, Vagelos College of Physicians and Surgeons, Columbia University, New York, NY 10032, USA; Gertrude H. Sergievsky Center, Vagelos College of Physicians and Surgeons, Columbia University, New York, NY 10032, USA; Department of Neurology, Vagelos College of Physicians and Surgeons, Columbia University, New York, NY 10032, USA

**Keywords:** biomarkers, neuroimaging, blood flow, Alzheimer’s disease, tau

## Abstract

Cerebrovascular dysfunction is associated with risk and progression of Alzheimer’s disease, but the extent to which it promotes Alzheimer’s pathophysiology is unclear. Understanding the relationship between cerebrovascular dysfunction and Alzheimer’s disease markers in midlife is critical to inform our understanding of the earliest manifestations of the disease and prevention strategies. We examined the association of cerebral blood flow with two biomarkers of tau pathophysiology, plasma phosphorylated tau 181 (p-tau181) concentrations and tau PET MK6240 standard uptake value ratio. This was a cross-sectional study of participants in the Offspring Study in upper Manhattan. Analyses included arterial spin labelling MRI, plasma p-tau181 concentration and 18F-MK-6240 tau PET data in the entorhinal cortex. Four hundred and fifty-nine participants (54.8 ± 10.8 years old, 63.3% women) had available MRI and plasma p-tau181 data, and 98 (60.4 ± 5.8 years old, 61.2% women) had additional tau PET data. Lower cerebral blood flow was associated with both higher plasma p-tau181 concentration and entorhinal cortex tau standard uptake value ratio. Higher plasma p-tau181 levels were associated with small clusters of lower regional cerebral blood flow, primarily in regions that correspond to sites of early Alzheimer’s disease pathology. Higher tau PET levels were associated with lower cerebral blood flow throughout the brain. These findings suggest that there is relationship between cerebral blood flow and indicators of tau pathophysiology in middle age that is likely bidirectional.

## Teaser

Houck *et al*. report that lower cerebral blood flow is associated with higher plasma phosphorylated tau 181 (p-tau181) concentrations and increased entorhinal cortex tau PET uptake in middle-aged individuals. These findings suggest a relationship between cerebrovascular function and tau pathology that is likely bidirectional.

## Graphical Abstract

**Graphical Abstract:**
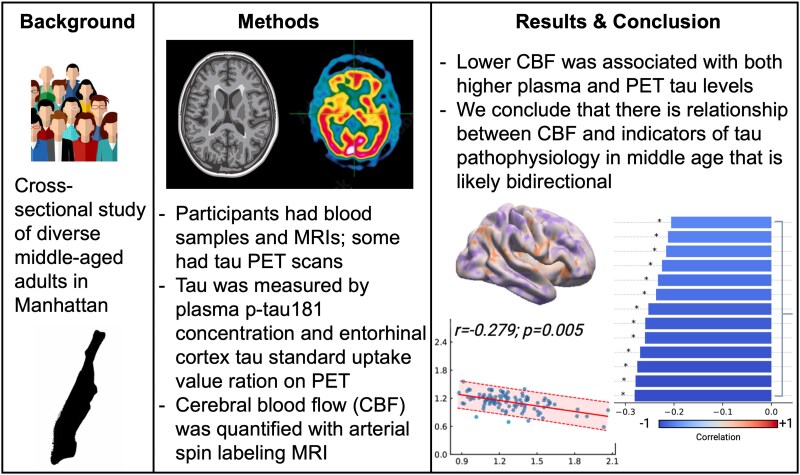

## Introduction

Reduction in cerebral blood flow (CBF) is a well-established characteristic of Alzheimer’s disease,^1^ but there is considerable debate about the role of cerebrovascular dysfunction in Alzheimer’s disease pathogenesis and disease progression.^2-5^ Emerging work suggests that in addition to reflecting common co-pathology, cerebrovascular changes may actively promote tau pathology in Alzheimer’s disease,^5^ perhaps mediated by blood–brain barrier dysfunction,^2^ and/or an inflammatory cascade.^3^ Animal models of small vessel cerebrovascular disease demonstrate that transient cerebral hypoperfusion may stimulate tau pathology independent of amyloidosis.^5^ Cerebrovascular dysfunction may additionally be downstream from emerging tau pathology, reflecting early ‘cell sickness’ prior to frank cell death or neurodegeneration.^4^

Assessment of either soluble or insoluble phosphorylated tau (p-tau) concentrations can contribute to the diagnosis of Alzheimer’s disease. Soluble p-tau biomarker levels can be detected up to two decades prior to the development of aggregated tau pathology,^6^ and these soluble p-tau levels peak and then plateau or even slightly decrease as the insoluble neurofibrillary tangle aggregates become detectable.^6,7^ Second-generation tau PET tracers, including ^18^F-MK-6240, accurately capture insoluble hyperphosphorylated tau-related neurofibrillary tangle pathology in symptomatic and asymptomatic adults across Alzheimer’s disease stages.^8,9^ These tracers demonstrate minimal off-target binding^10-12^ of tau from non-Alzheimer’s disease tauopathies, although they bind to neuromelanin, melanin-contacting cells and areas of haemorrhage.^13^ Similarly, new blood-based biomarkers can identify Alzheimer’s disease–related pathophysiology, including plasma concentrations of phosphorylated tau protein at threonine 181 (p-tau181). Elevated plasma p-tau181 concentrations are observed in Alzheimer’s disease, correspond with PET-based and autopsy-observed tau pathology and are helpful in distinguishing Alzheimer’s disease from other causes of dementia.^14^

In this study, we used pulsed arterial spin labelling (ASL) MRI to examine the association of regional CBF with both plasma and PET tau biomarkers in middle-aged adults from a racially and ethnically diverse community study. We focused on middle age for several reasons. Alzheimer’s disease pathophysiology can emerge decades before symptom onset,^15^ and it is therefore important to study the earliest manifestations of the disease to inform prevention strategies. Additionally, the correlates and determinants of Alzheimer’s disease–related pathophysiology may differ across the adult lifespan, yet there is very little information about the relationship between Alzheimer’s disease biomarker concentrations and brain health in middle age. The potential bidirectional association between markers of Alzheimer’s disease pathophysiology and cerebrovascular dysfunction in middle age may inform preventative strategies to mitigate the risk of future cognitive decline, in addition to shedding light on potential early determinants and consequences of Alzheimer’s disease biomarkers. We hypothesized that biomarkers reflecting increased tau pathology would be associated with decreased regional CBF.

## Materials and methods

### Participants

Participants came from the Offspring Study of Mechanisms for Racial Disparities in Alzheimer’s Disease.^16^ This is an ongoing observational study comprising the racially and ethnically diverse, middle-aged, adult children of participants in the Washington Heights Inwood Columbia Aging Project (WHICAP), a longitudinal, community-based study of cognitive aging and dementia in older adults residing in upper Manhattan, New York.^16^ Participants undergo a comprehensive neuropsychological examination and detailed psychosocial and medical history interview. As part of the neuropsychological assessment, participants are evaluated with the Selective Reminding Test (SRT),^17^ a test of list learning and memory. The SRT includes 6 trials to learn 12 words with selective reminding after the first trial, and following an approximately 15-min delay, participants are asked to recall as many of the 12 words from the learning trials. Eligible participants undergo advanced neuroimaging, including high-resolution MRI with ASL to assess cerebral perfusion. The majority of participants had blood samples collected for biomarker analysis, and a subset is also selected for PET imaging to quantify tau deposition.

### Plasma biomarkers

Plasma p-tau181 levels were measured with Single-molecule array (Simoa) technology on a fully automated Quanterix HD-X Analyzer. The p-tau181 measurements were quantified with the pTau-181 Advantage V2 Kit (catalogue #103714, Lot# 502691), adhering strictly to the manufacturer’s protocols. Quality control procedures included the assessment of intra-plate, inter-plate and intra-day variability. Dedicated plasma samples, included on each assay plate and analysed in duplicate, were used to monitor variability. Measurements with coefficients of variation of greater than 0.10 were removed from the analyses to ensure the reliability and accuracy of the biomarker measurements.

### Image acquisition and processing

Participants were scanned on a 3T MRI scanner (Siemens Prisma) equipped with a 64-gradient head coil at Columbia University between 2018 and 2023. For this study, T_1_-weighted and pulsed ASL (PASL) imaging sequences were used. The T_1_-weighted sequences were acquired with the following parameters: TE = 2.26 ms; TR = 2300 ms; TI = 900 ms; flip angle = 8 °; acquisition time = 5:21; resolution = 1 × 1 × 1 mm^3^; and dimensions = 256 × 256×192 mm^3^. PASL imaging was performed with the following parameters: TE = 13.36 ms; TR = 4600 ms; TI = 1990 ms; acquisition time = 5:36; bolus duration = 700 ms; resolution = 1.9 × 1.9 × 4 mm^3^; and dimensions = 128 × 128×36 mm^3^. The PASL sequence was repeated eight times in a four-labelled/four-control alternating pattern. A quantitative imaging technique for perfusion using a single-subtraction second version with a thin-section TI periodic saturation (Q2TIPS) was applied to all perfusion data.^18^ To quantify the CBF (mL/100 g/minute), motion correction was applied to each label-control image pair before performing subtraction. The resulting images were averaged to compute relative CBF, and absolute CBF values were derived using a consensus formula.^19,20^ CBF image quality was assessed using both quantitative and visual evaluations to ensure reliability. Quantitative assessment included signal-to-noise measurements between grey and white matter and mean frame-wise displacement for head motion, with established exclusion thresholds (SNR ≤ −2.3 or ≥3.4; motion > 0.319) based on published literature.^21^ We used FSL FMRIB tools for motion correction (mcflirt) and outlier detection (fsl_motion_outliers). Additionally, visual inspection was conducted to identify and reject low-quality ASL images with insufficient label-control pairs. Data from five participants were excluded due to having fewer label-control pairs than other participants, rendering their data inconsistent with the rest of the cohort.

Partial volume correction was subsequently applied to the CBF maps using the Müller–Gärtner (MG) method.^22^ We applied the FreeSurfer MG method through mri_gtmpvc, which leverages the cortical surface model to define precise grey/white matter boundaries. This correction models CSF contamination and reduces edge effects through 3 × 3 kernel regression, minimizing potential spill-in from adjacent structures. The corrected CBF maps were then registered to the T_1_-weighted images using a boundary-based inter-subject registration method with six degrees of freedom, which maximizes contrast at grey/white matter interfaces defined by the cortical surface reconstruction.^23^ The maps were then normalized to the whole brain mean, projected onto the brain cortical surface and smoothed with a full-width half-maximum of 5 mm, using tools in FreeSurfer v7.3.2. This surface-specific projection samples CBF values exclusively at the midpoint between pial and white matter surfaces, effectively isolating cortical signals from potential subcortical contamination. Cortical regions of interest (ROIs) were defined according to the Desikan–Killiany atlas, and left and right hemispheric parcellations of cortical parcellates were averaged to create single regions.^24^

A subset of the participants underwent tau ^18^F-MK-6240 PET imaging on a Siemens Biograph64 mCT/PET scanner (5 mCi, injected as slow bolus). Tau PET data were reconstructed with an iterative algorithm (3D OSEM) and corrected for radioactive decay, attenuation, scatter and random events and scanner normalization and dead time. Tau standard uptake value ratio (SUVR) in the entorhinal cortex was calculated using data 90–110 min post-injection with the inferior grey matter cerebellum serving as the reference region, as has been previously validated.^9,11,25^ The entorhinal cortex was chosen because it represents the earliest region of tau pathology in Alzheimer’s disease. PET image processing was performed with the SPM12 toolbox in MATLAB. To assess amyloid positivity, participants also received amyloid PET imaging with florbetaben, with a weighted average SUVR in Thal phase regions and amyloid positivity considered at >1.25 global SUVR.

### Statistical analyses

#### Surface-based correlation analyses

Surface-based correlation analyses were conducted using general linear models to investigate the relationship between regional CBF and plasma p-tau181 concentration and entorhinal cortex tau SUVR.^26^ The analyses used CBF data with a single offset (i.e. intercept) across all participants and included plasma p-tau181 concentration or entorhinal tau SUVR as a predictor and age as a covariate in separate models. We did not include amyloid as a covariate because prior research showed that the associations between tau PET and CBF are independent of amyloid pathology in a cohort across the Alzheimer’s disease continuum.^27^ Each model was subjected to three contrasts: one testing whether the intercept differs from zero, a second assessing the positive association of p-tau181 concentration or entorhinal cortex tau SUVR with CBF and a third evaluating the negative association of p-tau-181 concentration or entorhinal cortex tau SUVR with CBF. We conducted Z Monte Carlo simulations and applied a vertex-wise/cluster-forming threshold of 1.3 (*P* < 0.05) for cluster-wise correction for multiple comparisons. This approach identified significant clusters with *P*-values adjusted for both hemispheres using Bonferroni corrections.

#### Pairwise correlation analyses

Partial Pearson correlations, adjusted for age, were used to assess the association between CBF across cortical regions and plasma p-tau181 concentrations, as well as entorhinal cortex tau SUVR values. The analyses were conducted using JMP Pro 17.0 (SAS Institute Inc., Cary, NC, 1989–2023).

## Results

### Sample description

Table 1 displays demographic characteristics for all participants and for the subset with both ASL and tau PET data. At the time of analysis, 459 participants had both ASL MRI and plasma p-tau181 concentrations available; 98 participants had both ASL and tau PET imaging data, and of this subset, 68 also had plasma p-tau181 measurements. Only 9 of the 98 participants who underwent PET imaging were positive for amyloid. On the SRT, participants in the study learned a mean of 41.76 (SD = 10.60) on the learning trials and recalled a mean of 6.19 (SD = 2.68) words on the delayed recall trial, indicating that, on average, participants had normal memory function. The scores were normally distributed for both learning and delayed trials.

**Table 1:** Demographic characteristics of the participants

		Plasma p-tau181 group	Tau PET SUVR group
*N*		459	98
Age, mean (SD), years		54.8 (10.8)	60.4 (5.8)
Women, *n* (%)		291 (63.3)	60 (61.2)
Race and ethnicity	Latinx, *n* (%)	305 (66.4)	56 (57.1)
	Black, *n* (%)	118 (25.7)	32 (32.7)
	White, *n* (%)	29 (6.3)	10 (10.2)
	Other, *n* (%)	7 (1.5)	0 (0)
p-tau 181 concentration, mean (SD), pg/ml		1.76 (0.96)	1.77 (0.77)
Tau PET, mean (SD) SUVR in entorhinal cortex		N/A	1.29 (0.25)
Amyloid PET positivity, *n* (%)		N/A	9 (9.2)

### Relationship between cerebral blood flow and plasma p-tau181

**Figure 1:**
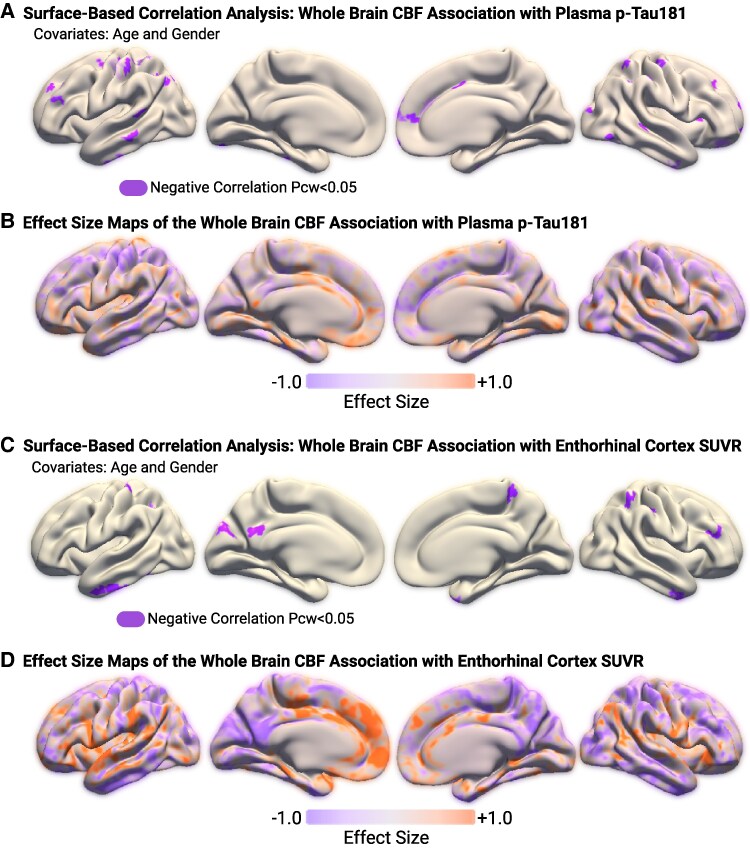
**Surface-based correlation analyses and effect size.** This figure illustrates the results of surface-based correlation analyses and their corresponding effect sizes. (**A**) Clusters of significant negative correlation between CBF and plasma p-tau181. (**B**) Effect size of the CBF and plasma p-tau181 correlation shown in **A**. (**C**) Clusters of negative correlation between CBF and tau SUVR in the entorhinal cortex. (**D**) Effect size of the CBF and tau SUVR correlation shown in **C**. The colour scale for effect size ranges from purple to orange, representing the magnitude of correlations in both directions. Darker colours indicate stronger correlations, while lighter colours represent weaker correlations. Grey areas in the template colour represent regions with zero or near-zero correlations. Sample sizes: plasma p-tau181 group (**A** and **B**): *n* = 459; tau PET SUVR group (**C** and **D**): *n* = 98.

**Figure 2:**
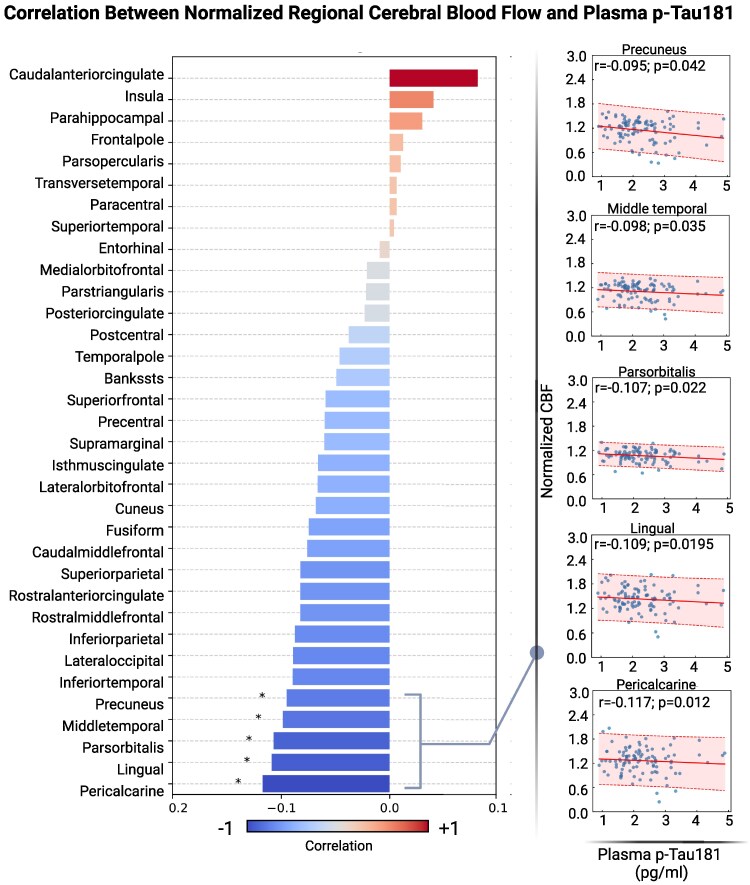
**Pairwise correlations between regional CBF and plasma p-tau181 concentration.** Bar plot (left) illustrating the pairwise Pearson correlations between CBF and plasma p-tau181 across brain regions (*n* = 459). The colour scheme indicates the directionality and strength of the correlations: blue represents negative correlations and red represents positive correlations, and the colour intensity corresponds to the strength of the correlation. Asterisks (*) denote statistically significant correlations (*P* < 0.05). Scatter plots (right) show individual data points for the statistically significant correlations.

Results of both the surface-based and pairwise correlation analyses comparing CBF and plasma p-tau181 are depicted in Table 2. For both analyses, lower CBF was associated with higher plasma p-tau181 concentration. In the surface-based correlation analyses, there were many clusters of negative correlation distributed throughout the brain, shown in Fig. 1A. The effect size map is depicted in Fig. 1B, with colour bars illustrating the magnitude of their respective correlations. In the pairwise correlation analyses, all significant associations between CBF and p-tau181 were also negative, and most other brain regions showed a negative trend but did not reach statistical significance (Fig. 2).

**Table 2:** The relationships between CBF and plasma p-tau181, surface-based analyses (top) and pairwise correlation analyses (bottom)

p-tau 181 analyses					
	Brain region	Cluster size (mm^2^)	PCC	CWP	CI for CWP (95%)
Surface-based analyses	Middle temporal	94.6	−0.425	0.0002	(0.0001, 0.0004)
	Inferior temporal	136.8	−0.410	0.0002	(0.0001, 0.0004)
	Inferior temporal	97.0	−0.405	0.0002	(0.0001, 0.0004)
	Precentral	127.9	−0.400	0.0002	(0.0001, 0.0004)
	Superior parietal	174.4	−0.399	0.0002	(0.0001, 0.0004)
	Rostral middle frontal	99.6	−0.305	0.0002	(0.0001, 0.0004)
	Postcentral	834.0	−0.301	0.0002	(0.0001, 0.0004)
	Rostral middle frontal	229.9	−0.298	0.0002	(0.0001, 0.0004)
	Postcentral	84.7	−0.105	0.0006	(0.0001, 0.0004)
	Postcentral	84.1	−0.101	0.0008	(0.0004, 0.0014)
	Superior parietal	72.0	−0.098	0.0044	(0.0032, 0.006)
	Precentral	65.8	−0.091	0.0106	(0.0087, 0.012)
	Inferior parietal	63.3	−0.112	0.0148	(0.013, 0.020)
	Inferior parietal	61.7	−0.112	0.0177	(0.015, 0.020)
	Lateral occipital	59.0	−0.098	0.0270	(0.024, 0.029)
	Rostral middle frontal	58.4	−0.105	0.0298	(0.026, 0.030)
	Banks of the superior temporal sulcus	58.2	−0.120	0.0314	(0.009, 0.030)
	Inferior parietal	58.1	−0.107	0.0314	(0.008, 0.030)

	Brain region	*R*	CI for *R* (95%)	*P*-value
Pairwise correlation analyses	Pericalcarine	−0.117	(−0.207, −0.026)	0.012
	Lingual	−0.109	(−0.199, −0.018)	0.020
	Pars orbitalis	−0.107	(−0.197, −0.016)	0.022
	Middle temporal	−0.099	(−0.188, −0.007)	0.035
	Precuneus	−0.095	(−0.185, −0.004)	0.042

All statistically significant relationships for the plasma p-tau181 analyses are depicted. The top section shows the surface-based analyses, arranged by cluster-wise *P*-value (CWP). All significant relationships are negative, with multiple clusters sometimes identified within a brain region. In the surface-based analyses, confidence intervals are reported around *P*-values because these analyses rely on permutation-based methods or simulations. The bottom section shows the pairwise correlation analyses, arranged by *P*-value. All significant relationships here are also negative; brain regions were analysed as a whole, so it was not possible for brain regions to have multiple representations, unlike in the surface-based analyses. Confidence intervals around the correlation coefficient are reported. Brain regions are defined according to the Desikan–Killiany atlas.

PCC, partial correlation coefficient; CWP, cluster-wise corrected *P*-value; CI, confidence interval; *R*, Pearson correlation coefficient.

### Relationship between cerebral blood flow and entorhinal cortex tau PET standardized uptake value ratio

**Figure 3:**
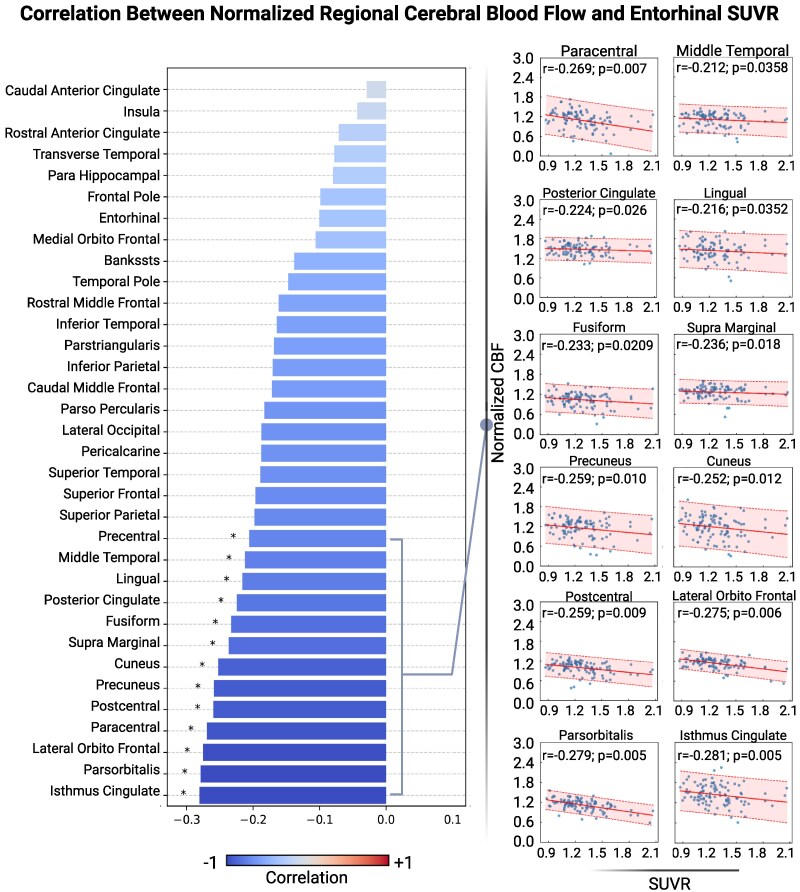
**Pairwise correlations between regional CBF and entorhinal cortex tau SUVR.** Bar plot (left) illustrating the pairwise Pearson correlations between CBF and entorhinal cortex tau SUVR across brain regions (*n* = 98). The *x*-axis represents the Pearson correlation coefficient values, while the *y*-axis displays the brain regions. The colour scheme indicates the directionality and strength of the correlations: blue represents negative correlations and red represents positive correlations, and the colour intensity corresponds to the strength of the correlation. Asterisks (*) denote statistically significant correlations (*P* < 0.05). Scatter plots (right) show individual data points for the statistically significant correlations.

Results of both the surface-based and pairwise correlation analyses comparing CBF and entorhinal cortex tau PET SUVR are depicted in Table 3. For both analyses, lower regional CBF was associated with higher entorhinal cortex tau SUVR. In the surface-based correlation analyses, there were several clusters of negative correlation distributed throughout the brain, shown in Fig. 1C. The effect size map is depicted in Fig. 1D. In the pairwise correlation analyses, all significant associations between CBF and entorhinal cortex tau SUVR were also negative, similar to the pattern seen in the CBF-p-tau181 analyses. All other regions showed a negative trend but did not reach statistical significance (Fig. 3).

**Table 3:** The relationship between CBF and entorhinal cortex tau PET SUVR, surface-based analyses (top) and pairwise correlation analyses (bottom)

Entorhinal cortex tau PET SUVR analyses					
	Brain region	Cluster size (mm^2^)	PCC	CWP	CI for CWP (95%)
Surface-based analyses	Superior parietal	416.9	−0.312	0.0002	(0.0001, 0.0004)
	Cuneus	160.9	−0.291	0.0016	(0.0012, 0.0021)
	Inferior temporal	153.6	−0.320	0.0024	(0.0012, 0.003)
	Inferior temporal	148.1	−0.315	0.0034	(0.0029, 0.039)
	Postcentral	117.9	−0.330	0.0220	(0.018, 0.025)
	Inferior temporal	115.7	−0.314	0.0244	(0.017, 0.026)
	Isthmus cingulate	115.5	−0.274	0.0246	(0.023, 0.027)
	Inferior parietal	114.3	−0.296	0.0264	(0.024, 0.027)

	Brain region	*R*	CI for *R* (95%)	*P*-value
Pairwise correlation analyses	Isthmus cingulate	−0.281	(−0.454, −0.087)	0.005
	Pars orbitalis	−0.279	(−0.452, −0.085)	0.005
	Lateral orbitofrontal	−0.276	(−0.449, −0.082)	0.006
	Paracentral	−0.270	(−0.444, −0.075)	0.007
	Postcentral	−0.260	(−0.436, −0.065)	0.010
	Precuneus	−0.259	(−0.435, −0.064)	0.010
	Cuneus	−0.253	(−0.430, −0.057)	0.012
	Supramarginal	−0.237	(−0.416, −0.040)	0.019
	Fusiform	−0.233	(−0.412, −0.036)	0.021
	Posterior cingulate	−0.225	(−0.405, −0.028)	0.026
	Lingual	−0.216	(−0.398, −0.019)	0.033
	Middle temporal	−0.212	(−0.394, −0.015)	0.036
	Precentral	−0.206	(−0.389, −0.008)	0.042

All statistically significant relationships for the entorhinal cortex tau PET SUVR analyses are depicted. The top section shows the surface-based analyses, arranged by cluster-wise *P*-value (CWP). All significant relationships are negative, with multiple clusters identified within the inferior temporal lobe. In the surface-based analyses, confidence intervals are reported around *P*-values because these analyses rely on permutation-based methods or simulations. The bottom section shows the pairwise correlation analyses, arranged by *P*-value. All significant relationships here are also negative; brain regions were analysed as a whole, so it was not possible for brain regions to have multiple representations, unlike in the surface-based analyses. Confidence intervals around the correlation coefficient are reported. Brain regions are defined according to the Desikan–Killiany atlas.

PCC, partial correlation coefficient; CWP, cluster-wise corrected *P*-value; CI, confidence interval; *R*, Pearson correlation coefficient.

## Discussion

Lower CBF was associated with both increased plasma p-tau181 concentration and entorhinal cortex tau SUVR in middle-aged adults from a racially and ethnically diverse community sample. This negative association between regional CBF and measures of tau pathophysiology is consistent with prior research that describes cerebrovascular dysfunction and CBF reduction as a well-established characteristic of Alzheimer’s disease in clinical populations.^1,28-30^

In surface-based analyses, we saw numerous clusters of negative association between CBF and plasma p-tau181, as well as fewer, but still negative, clusters of association between CBF and entorhinal cortex tau SUVR. When evaluating ROIs more generally with the pairwise correlation analyses, we found that entorhinal cortex tau SUVR was more strongly and consistently associated with CBF than plasma p-tau181. As seen in the surface-based correlation analyses, the pattern of distribution of decreased regional CBF appears in multiple regions, with associations of plasma p-tau181 concentrations and tau PET with CBF converging anatomically particularly in the inferior temporal lobe, the postcentral gyrus, the inferior parietal lobe and the superior parietal lobe, areas typically associated with Alzheimer’s disease pathology.

In the plasma p-tau181 pairwise correlation analyses, negative associations were observed more globally, throughout the frontal, temporal, parietal and occipital lobes, specifically in the pericalcarine cortex, lingual gyrus, pars orbitalis, middle temporal lobe and precuneus. Four of these five regions overlap with the negative associations seen in the entorhinal cortex tau SUVR pairwise correlation analyses, showing convergence in the association of CBF with two indices of tau pathophysiology. In the entorhinal cortex tau SUVR pairwise correlation analyses, there were many other regions throughout the brain with strong negative associations in the frontal, temporal, parietal, occipital and limbic lobes. Thus, while both the surface-based and pairwise correlation analyses showed consistently negative relationships of CBF with both plasma p-tau181 and tau PET, the regions did vary, indicating small clusters of voxels throughout the brain where regional CBF is associated with tau pathophysiology that are obscured with larger regional analyses. Conversely, larger brain regions showed strong relationships in the pairwise analyses without corresponding clusters.

Interestingly, we did not find a statistically significant association specifically between entorhinal cortex tau SUVR and entorhinal cortex CBF, although we did observe reliable relationships with CBF in adjacent regions, like the middle temporal and lingual gyri. There may be methodological and physiological considerations for this observation. Arterial spin labelling with background suppression and smaller slice thickness may better capture CBF in small ROIs, and ASL with time encoding may be more sensitive to earlier changes. Additionally, participants in this study were, on average, middle age. The large dynamic range of MK6240 may be sufficient to detect variability in entorhinal tau SUVR, but CBF reductions in these regions may be too subtle to detect in this age stratum.

Combined, the surface-based and pairwise analyses suggest that the impact of cerebrovascular dysfunction on tau pathology (and/or vice versa) may be simultaneously overlapping with, and regionally distinct from, stereotypical patterns that are observed in Alzheimer’s disease. Of note, our analyses examined the relationship between CBF and measures of tau pathology, without respect to disease status, risk or severity. Thus, it is possible that there is a reliable relationship between vascular dysfunction and tau pathology in areas that are distinct from Alzheimer’s disease–associated regions in individuals without frank disease.

Prior studies showed that while plasma p-tau levels are associated with tau PET levels,^31^ biofluidic p-tau levels plateau prior to tau PET levels, suggesting that p-tau represents the early, dynamic process of phosphorylation and secretion from the neuron, and tau SUVR could reflect later-stage neurofibrillary tangles.^7^ These differences suggest that there is a relationship between CBF and tau pathophysiology, which may be bidirectional and multifactorial, although the exact mechanisms are not yet well established. On the one hand, decreased CBF contributes to tau pathology, likely through a complex interplay of mechanisms involving brain hypoxia, impaired protein clearance, neuroinflammation and oxidative stress.^5,32^ Typically, cerebral autoregulation strictly regulates CBF to maintain consistent oxygen delivery, and thus lowered CBF inherently leads to decreased available oxygen to brain tissues.^33^ HIF1A is a protein synthesized in the setting of decreased oxygen, and its production is hypothesized to downregulate neuroglobin, an oxygen binder, triggering the generation of free radicals and activating microglia-derived neuroinflammatory factors, including mitogen-activated protein kinase, resulting in tau hyperphosphorylation.^32^ Chronic cerebral hypoperfusion may also decrease brain glucose metabolism, leading to downregulation of tau O-GlcNAcylation, resulting in hyperphosphorylation via tau disruption in microtubules.^34^ This cortical and hippocampal hyperphosphorylation is seen in both Alzheimer’s disease and wild-type mice that do not develop amyloidosis.^5,35^ Another mechanism by which decreased CBF might contribute to tau phosphorylation is via the blocking of unfolded protein response, which would typically activate in response to tau misfolding in order to restore normal function and degrade misfolded proteins.^36^ The mammalian target of rapamycin (mTOR) is an ubiquitous kinase that mediates the synthesis and aggregation of p-tau. Animal models of cerebral ischaemia and oxygen deprivation show hyperactivation of mTOR pathways, contributing to excessive tau phosphorylation.^37^ In mice displaying brain hypoperfusion, mTOR inhibition can restore CBF.^38^ And lastly, impairment in neurovascular function can lead to reduced clearance of tau from the brain, particularly via the glymphatic system.^39^

On the other hand, tau pathology may directly contribute to decreased CBF, and several complex mechanisms have been proposed.^4,40^ Tau-overexpressing mice develop abnormal blood vessel morphologies, suggesting that neurofibrillary tangles harm the integrity of the cortical microvasculature.^41^ Tau oligomers accumulate in the perivascular space of intraparenchymal brain vessels, which may contribute to vessel stiffness, elastin degradation and impaired drainage.^42^ Numerous studies linked tau with local neuroinflammation.^43-45^ Mechanistically, in a multifaceted process, tau may propagate to the cellular components of the neurovascular unit, inducing harmful neuroinflammatory and mitochondrial effects, and furthermore, caspase-3 induces tau cleavage, causing toxic effects on neurovascular cells.^4,40^ Thus, early vascular changes likely contribute to tau pathology, and tau pathology may in turn manifest as vascular dysfunction, suggesting a potential positive feedback loop.

Our study is unique in that we evaluated markers of tau pathology in a community-based sample of middle-aged adults, rather than a clinical or elderly cohort, which could provide insights into early pathophysiological changes. A negative relationship between CBF and tau PET levels in cognitively unimpaired adults was previously observed, but this study only evaluated ASL in the temporal lobe and included participants who were primarily non-Hispanic white and older.^46^ Our findings suggest that, even in the absence of frank clinical disease, cerebrovascular dysfunction may contribute to tau pathology, potentially initiating a cycle of worsening cerebrovascular health and progressive tau pathology. The differences we see with stronger surface-based cluster relationships between CBF and p-tau181 compared to the stronger regional relationships between CBF and tau PET may reflect the nuances of a bidirectional relationship between CBF and tau pathology; small local cerebrovascular injury could contribute more to plasma p-tau181 concentration, whereas insoluble neurofibrillary tangle deposits themselves could contribute more to globally decreased CBF. These results align with prior studies of tauopathy showing that local cerebrovascular changes, such as endothelial senescence, are associated with increased tau pathology while tau accumulation along vascular segments is related to neurofibrillary tangle formation,^47^ with large-scale CBF alterations representing later stages of disease.^48^

We interpret our findings in the context of several limitations. First, the cross-sectional design prevents causal inferences and limits the ability to observe dynamic cerebrovascular changes over time. Longitudinal studies would be necessary to determine the temporal dynamics of the associations that we found. Second, MRI-ASL has limitations in resolution and volumes, which may affect the precision of CBF measurements, particularly in small regions like the entorhinal cortex, even with partial volume correction. Finally, there may be other factors influencing CBF and tau pathology that we did not evaluate, including other aspects of cardiovascular health and APOE genotype, which could be considered for future studies.

In conclusion, we found that lower CBF in middle-aged adults is associated with increased plasma p-tau181 concentration levels and entorhinal cortex tau SUVR levels.

## Data Availability

The data from the Offspring Study are available upon written request and review by the study investigators.
